# Analysis of COVID-19 vaccination experience of the Los Pastos, Wounaan, and Misak Misak indigenous peoples from Bogotá, Colombia

**DOI:** 10.1371/journal.pgph.0004870

**Published:** 2025-07-11

**Authors:** Sandra Vargas-Cruz, Miguel Baquero-Acuña, Camila Bautista, Juan Castro-Caro, Nicol Espejo, Pedro Ruiz Mateus, Irene Parra-García

**Affiliations:** 1 Faculty of Medicine, Universidad El Bosque, Bogotá, Colombia; 2 Humanities Department, Universidad El Bosque, Bogotá, Colombia; PLOS: Public Library of Science, UNITED STATES OF AMERICA

## Abstract

The coronavirus disease 2019 (COVID-19) pandemic was recognized as a public health crisis closely linked to socioeconomic and cultural factors. Vaccination is a long-term solution for COVID-19; however, access to vaccines has been hindered by geographic, cultural, and socioeconomic barriers as well as distrust in the health system—particularly among populations that have experienced historical inequities, such as indigenous peoples. This study aimed to analyze the COVID-19 vaccination experiences of the Misak Misak, Wounaan, and Los Pastos indigenous peoples in Bogotá, Colombia. This was a sequential explanatory mixed-methods study, with the quantitative phase followed by the qualitative phase. We conducted emistructured interviews, 9 sharing circles, and 85 household surveys using a triangulation approach. Results showed that the Los Pastos people had the highest vaccination rate (97.3%), followed by the Misak Misak people (85.2%); the Wounaan people had the lowest vaccination rate (38.5%). The main reason for vaccination among the Misak Misak and Wounaan was that it was mandated by their workplaces or educational institutions. For the Los Pastos, the main reason for vaccination was to protect themselves and their environment. The main reasons for not getting vaccinated included distrust of vaccines, although there were no geographic access barriers. This study revealed varying vaccination rates among indigenous populations living in urban areas, possibly associated with factors such as infodemic, previous distrust of Western health services, and preference for ancestral medicine as an alternative for COVID-19 prevention.

## Introduction

SARS-CoV-2 was identified in December 2019 and rapidly spread worldwide, leading to its declaration as a pandemic on March 11, 2020 [1]. This led to a global health crisis, as the virus is responsible for severe acute respiratory syndrome with varying symptoms. By April 12, 2024, the global death toll had reached 7,010,681 [2], with 2,950,808 deaths in the Americas by August 2023 [3] and 142,727 deaths in Colombia by June 2023 [4].

The coronavirus disease 2019 (COVID-19) pandemic was recognized as a public health crisis closely linked to social factors. It was marked by its strong connection with socioeconomic factors, as observed across various regions and populations [5]. Although the virus is considered to affect all socioeconomic groups equally, previous studies have suggested a relationship between biological and socioeconomic factors and increased mortality among migrant populations, historically racialized populations, precarious workers, and other vulnerable social groups [6,7].

All these populations were disproportionately affected because they could not comply with isolation measures, thus increasing their exposure. Moreover, they often lack the resources to access the healthcare system, or when they do, they face linguistic and cultural barriers as well as discrimination. Subsequently, this contributes to the barriers to COVID-19 vaccination [8–10]. In the case of historically racialized populations, discrimination and inequities have resulted in lower vaccination coverage than that in the general population [11]. The disparities persisted even during global health emergency such as COVID-19 [12]. Consequently, studies have been conducted on the enablers of and barriers to COVID-19 vaccination among these groups, including indigenous populations worldwide. In the United States and Canada, most communities harbored distrust toward vaccines and their effectiveness, encountered barriers while accessing services, experienced unequal treatment, and faced linguistic gaps that hindered the reception and comprehension of information [13,14].

A similar situation was observed in Latin America. Studies conducted in Guatemala, Brazil, and Paraguay revealed lower COVID-19 vaccination rates among indigenous peoples than in general population [8,15–17]. This was explained by the lack of quality information, geographical barriers, language communication barriers, and distrust in the vaccine development process [8]. Therefore, vaccination of the indigenous population is considered a multifactorial issue, with sociocultural factors playing a crucial role in achieving high coverage rates. As evidenced by the successful vaccination efforts among indigenous populations, such as Alaska Natives, high SARS-CoV-2 vaccination rates were achieved through culturally informed policies and the active involvement of these communities in the process [18].

In Colombia, by January 2024, 174.47 doses of COVID-19 vaccine were administered per inhabitants, ranking 13th in Latin America [19]. Regarding COVID-19 vaccination among indigenous populations, most studies have focused on communities within their territory of origin, which are often remote rural areas. This suggests the common geographical barriers to access as well as a clear rejection of the vaccine, primarily due to distrust in Western medicine and, as observed in the general population, fear of adverse effects [20,21].

As described above, the few studies conducted on this topic in Latin American indigenous populations focus on the communities’ territories of origin, which tend to be rural. This approach is pertinent given that these areas are inhabited by most of the native peoples of the region; however, the indigenous people residing in the city were not considered in most cases due to migration from their territories of origin because of social conflicts or the search for better opportunities, making them a migrant and historically racialized population [22–24].

Understanding the experiences of these populations is essential to ensure the protection of their rights, particularly their right to health. In addition to providing healthcare services to this population during the COVID-19 pandemic, understanding the vaccination process offers insights into how the healthcare system and its discourses interact with the population, particularly in terms of intersectionality and social markers [25]. This study aimed to examine the relationship by focusing on the dynamics of the COVID-19 vaccination process among indigenous populations in an urban setting. Therefore, this study analyzed COVID-19 vaccination experiences of the Misak Misak, Wounaan, and Los Pastos indigenous peoples in the city of Bogotá.

## Materials and methods

### Background

In Colombia, vaccination against COVID-19 began on February 17, 2021, with the country’s national immunization plan. The process was divided into five stages of prioritization according to different risk factors. Indigenous people were prioritized in stages 1 and 2. Vaccination was performed by national and regional health authorities. In Bogotá, the District Health Secretariat (local health authorities) established more than 30 vaccination centers in schools, shopping malls, public squares, and coliseums across the city, with continuous support and participation from health insurance companies, which also provided vaccination for their affiliated populations [26,27]. These centers operated up to 12 h a day (from 8 a.m. to 8 p.m.), with some locations extending their working hours. Initially, an appointment was required; however, during the final months, the centers attended on demand. By August 1, 2021, 5 million vaccines 129 had been administered in Bogotá [27]. In 2021, 11,519,140 doses of the vaccine were administered in the city, and this number was 4,534,532 in 2022 [26]. The city’s total population in 2022 was 7.9 million [28].

In 2021, the District Health Secretariat sought to vaccinate indigenous communities living in Bogotá to combat COVID-19 through vaccination campaigns coordinated with community leaders [29]. Although the Secretariat reported having a “differential approach” for these campaigns, it was not possible to determine exactly what this approach involved. These vaccination campaigns were conducted similarly to other campaigns, with the only distinction being that they were coordinated in advance with the indigenous leaders. Notably, the same prioritization system was maintained throughout the country, with indigenous communities included in the first two stages. In addition, it was through the leaders of each community that individuals were registered in the databases for prioritization. Data from the Secretariat indicate that the Los Pastos indigenous community had the second-highest participation in the COVID-19 vaccination process, with specific vaccination days scheduled for them [30].

### Study design

This study employed a sequential explanatory mixed-methods design, with a quantitative phase followed by a qualitative phase. The objective of the qualitative phase was to explain the quantitative findings and expand the research [31].

### Participant selection

Participant selection was performed in the city of Bogotá, capital of Colombia, located in the center of the country and on the eastern mountain range of the Andes [32]. The city of Bogotá was home to 19,063 (0.27%) indigenous people in 2018 [33]. Participants in this study were members of the three indigenous communities located in the city of Bogotá and were grouped and organized through their own organizations, similar to a indigenous tribal councils, called *Cabildos*, as described below:

a) The *Cabildo Indígena Wounaan nonam de Bogotá DC* groups the Wounaan indigenous people. By 2018, 278 inhabitants of the Wounaan population resided in Bogotá, most of whom lived in the Ciudad Bolivar locality [33]. Originally from the department of Chocó, they were individual and collective victims of the long-standing Colombian armed conflict (disputed by armed groups such as the guerrillas, paramilitary groups, and Military Forces of Colombia); drug trafficking groups; and illegal mining, as they have been displaced from their territories [24].
b) *The Autoridad Ancestral Misak Misak Nukotrak Bogotá DC* is the organization of the Misak Misak indigenous people from the departments of Cauca and Huila [24]. They migrated to Bogotá because of land production issues and are individual and collective victims of the armed conflict. In the 2018 census, there were 358 inhabitants of the Misak Misak population in Bogotá, mainly in the locality of Fontibón [33].
c) *The Cabildo indígena de Los Pastos in Bogotá DC* groups the Los Pastos people, who were originally located in the departments of Putumayo, Nariño, and Valle del Cauca. They migrated to Bogotá DC due to the armed conflict and lack of opportunities in their territory [24]. They are one of the peoples with the largest presence in the city of Bogotá, with a population of 503 inhabitants as of 2018 [33].

IP and SP have been working with those communities since 2017; however, the recruitment period for this specific study started on August 1, 2023, and ended on February 29, 2024.

#### Phase 1: quantitative phase.

A household survey was conducted to investigate aspects related to the number of people infected by SARS-CoV-2, vaccine administration, number of administered doses, and reasons for getting or not getting vaccinated. To estimate the sample size, the following was considered regarding the community: (a) to contact as few households as possible to avoid overburdening the population, considering that they are frequently approached for research purposes; and (b) the constant mobility of households between their territories of origin and Bogotá may reduce the number of *Cabildo* members living in the city during the pandemic.

Based on these factors, we considered a proportion of 50%, a confidence level of 80%, a margin error of 10%, and the number of households belonging to the *Cabildo*. Accordingly, the estimated sample was 37 for Los Pastos, 26 for Wounaan, and 27 for Misak Misak households. A stratified random sampling based on the number of persons per household was performed using household databases of each of the Misak Misak, Wounaan, and Los Pastos *Cabildos* in Bogotá. Inclusion criteria for the survey were having lived in Bogotá for ≥6 months during 2020 and/or 2021, being part of the *Cabildo* at the time of collection and being ≥18 years of age.

The survey form was developed by the researchers and reviewed in Spanish by the entire team, including one indigenous researcher from each of the participating indigenous groups. This included addressing both conceptualization and operational components. Moreover, the same team conducted a pilot test, and the form was reviewed by two experts (a physician and an anthropologist). The indigenous researchers collected information, each from their own community, through telephone calls and Google forms.

Statistical methods used for data analysis were descriptive statistics for quantitative variables and frequencies for qualitative variables. Biases were controlled using measures such as validation of the instrument, standardization, and parameterization using a digital data collection tool. Indigenous researchers acted as surveyors; they conducted a pilot test with two households of their own community (totaling six pilots). The final version of the survey was used to develop a manual for completion, and surveyors were trained in advance and monitored to ensure accurate data collection and response accuracy.

#### Phase 2: qualitative phase.

Three researchers (IP, SV, and a medical intern) conducted 20 face-to-face semistructured interviews. The indigenous researchers helped create an interview guide, which included topics such as vaccination logistics, vaccination process, access barriers, reasons for vaccination, opinions, and experiences about vaccination as well as alternatives to vaccination. Interviewees were selected with the assistance of indigenous authorities to identify those who played a leadership role—such as ancestral health practitioners and healers, authorities, leaders, and allies—during the pandemic and to determine variability in factors such as sex, age, and role within the *Cabildo*.

Additionally, a community gathering convened by the *Cabildo* was held for each respective *Cabildo*. These collective activities were conducted while respecting and honoring the community’s customs and traditions, including a harmonization sacred ritual held by ancestral healers and traditional food sharing. During these events, group interviews were conducted under sharing circles dynamics, enabling researchers to understand the community perspective through an essential practice rooted in the oral traditions of Colombia’s indigenous peoples [34]. Three sharing circles—one for each *Cabildo*—were held at each meeting to reflect on the pandemic. Participants engaged in conversations characterized by active listening, considering each other as equals, and mutual respect [35]. The total number of participants was 94 (36 men and 58 women), with the following characteristics:

• Misak Misak: 40 participants; 17 men and 23 women; 16, 14, and 10 participants in each sharing circle.

- Los Pastos: 29 participants; 10 men and 19 women; 10, 8, and 11 participants in each sharing circle.
- Wounaan: 25 participants; 9 men and 16 women; 8, 9, and 8 participants in each sharing circle.

**Fig 1:**
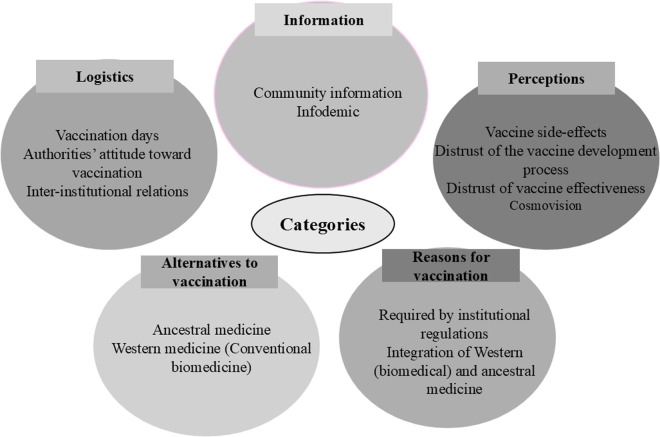
Categories and codes used for analyzing interviews and sharing circles on COVID-19 vaccination among indigenous peoples living in Bogotá.

For processing purposes, all interviews and sharing circles were recorded and subsequently transcribed verbatim. Data analysis was performed using the interpretative phenomenological approach, which aims to understand the meanings associated with personal experiences [36]. This process consisted of the following stages:

1. Reflective data collection: After the group meetings, researchers used reflective field notes and shared their experiences.
2. Transcription: Interviews and sharing circles were transcribed individually into Word documents.
3. Data reduction: Transcripts were revised to obtain comprehensible narratives. Nonverbal expressions or pauses within the transcripts were not considered; however, participants’ words were retained to organize and present the interviews as literally as possible and to preserve the meaning of each narration.
4. Thematic analysis: The interviews and sharing circles were reviewed by each researcher. An initial categorization was proposed based on the literature review and the objective of the study. However, new categories and subcategories emerged during the process. A coding manual identifying five categories was developed: logistics, information, perception, reasons for vaccination, and alternatives to vaccination. Each category is subdivided into the codes presented within each circle (Fig 1).

Data were coded (MB, CB, JC, NE, and PR) based on the guidance provided by IP and SV using the Atlas.ti version 9 software. To ensure credibility and validity of the qualitative component, Lincoln’s criteria [31] of confirmability, transferability, consistency, and credibility were applied. Data saturation was verified through the coding process, which included follow-up of a similar frequency of the codes and the varied origins of the stories from multiple interviews and sharing circles. In the final stage of the study, quantitative and qualitative data were analyzed to pool the results and to reach integrated conclusions. Thus, we developed a joint presentation focusing on how qualitative results coincided with, improved, or clarified quantitative results.

### Ethical considerations

This research was approved by the Institutional Research Ethics Committee of Universidad El Bosque, with approval record no. 008–2022. Each participant signed an informed consent form that ensured 253 voluntary participation and respect for their community’s customs and traditions. The research process was adapted to include sharing circles and the participation of indigenous researchers throughout. All of the above was carried out in consultation with the traditional authorities of the *Cabildos*, who approved the initial execution of the project, participated in its activities, and were kept informed throughout its development.

## Results

The primary characteristics of the respondents are presented below. In the Misak Misak indigenous people, 27 surveys were conducted; the average age of the participants was 39.6 ± 11.6 years, and 16 respondents (59.2%) were men. Free union was the predominant marital status (n = 18; 66.7%). The locality with the highest number of respondents was Fontibón with 23 respondents (85.2%). Regarding participants’ educational level, 12 (44.4%) had completed high school. In terms of health affiliation, 23 respondents (85.1%) were affiliated with a health insurance regime, with 15 (55.6%) being part of the contributory system and 8 (29.6%) of the subsidized system.

In the Wounaan people residing in Ciudad Bolivar, a total of 26 surveys were conducted. Participants’ average age was 37.9 ± 11.4 years, and 19 respondents (73%) were men. Free union was the predominant marital status (n = 20; 76.9%) participants. Regarding education, 10 (38.5%) respondents had completed high school. In terms of health insurance, 26 (100%) were affiliated with a regime, and most were in the subsidized regime (20 [76.9%] participants).

Finally, a total of 37 surveys were conducted with the Los Pastos people. Respondents’ average age was 273 38.3 ± 11.3 years, 23 (62.2%) were women, and the most common marital status was single (n = 24; 64.9%), followed by married (n = 8; 21.6%). Regarding the locality, the highest number of respondents lived in Suba, accounting for 7 (18.9%) respondents, followed by the localities of San Cristóbal and Mártires with 6 (16.2%) respondents each. The number of participants with a high school diploma was 15 (40.5%), followed by basic primary education in 8 (21.6%). All respondents of the Los Pastos people were affiliated with a health care system, with 25 (67.6%) being affiliated with the contributory system.

The results of the survey showed that the proportion of respondents among whom a household member contracted COVID-19 was high in the Misak Misak households with 22 (81.5%) individuals, followed by the Wounaan individuals with 16 (61.5%) individuals, and finally Los Pastos with 12 (32.4%) individuals. Additionally, higher percentages of vaccination were observed among the respondents of the Los Pastos people, with 36 respondents having received at least one dose of a COVID-19 vaccine (97.3%), followed by the Misak Misak people with 23 respondents (85%) and the Wounaan people with 10 respondents (38.5%) (Table 1).

**Table 1:** Results of the survey on infection rates and COVID-19 vaccination in Misak Misak, Wounaan, and Los Pastos peoples from Bogotá.

Question	Misak Misak		Wounaan		Los Pastos		Total	
Have you or any member of your household contracted COVID-19?	n	%	n	%	n	%	n	%
No	5	18.5	8	30.8	25	67.6	38	42.2
Does not know/does not answer/does not remember	0	0	2	7.7	0	0	2	2.2
Yes	22	81.5	16	61.5	12	32.4	50	55.6
Total	27	100.0	26	100.0	37	100.0	90	100.0
Have you received any COVID-19 vaccine doses?								
No	4	14.8	16	61.5	1	2.7	21	23.3
One dose	9	33.3	4	15.4	11	29.7	24	26.7
Two doses	5	18.5	4	15.4	24	64.9	33	36.7
Three doses	9	33.3	2	7.7	1	2.7	12	13.3
Total	27	100.0	26	100.0	37	100.0	90	100.0
Why did you get vaccinated?								
My family/job/school requires me to do it	19	82.6	3	30	3	8.3	25	36.2
To return to work, school, social or community activities, or to travel	3	13.0	6	60	13	36.1	22	31.9
Other (I was encouraged by others, to protect my health or the health of my family, friends and/or community)	1	4.4	1	10	20	55.6	22	31.9
Total number of vaccinated individuals	23	100.0	10	100.0	36	100.0	69	100.0
Why did you refuse to vaccinate?								
Because it goes against my beliefs	3	75	13	81.3	1	100	17	81.0
Other (the vaccine caused disease, was not safe, fear of needles)	1	25	3	18.7	0	0	4	19.0
Total number of unvaccinated individuals	4	100.0	16	100.0	1	100.0	21	100.0
	**x̅**	SD	**x̅**	SD	**x̅**	SD	**x̅**	SD
Number of household members NOT vaccinated against COVID-19	5.4	1.3	2.6	4.25	0.03	0.16	2.7	1.9
Number of household members vaccinated against COVID-19	3.6	3.5	2.6	1.8	2.3	1.2	2.8	2.2

Regarding the reasons for vaccination, the responses were similar among the Misak Misak and Wounaan people, for whom the primary reason was obligation by their jobs, educational institutions, or community, with 19 respondents (83%) from the Misak Misak and 3 (30%) from the Wounaan. For Los Pastos, the main reason was to protect their own health or that of their community, as reported by 20 respondents (56%). Regarding the reasons for not getting vaccinated, all three communities agreed that it was against their beliefs, with 3 respondents (75%) from the Masak Misak, 13 (81.2%) from the Wounaan, and 1 (100%) from the Los Pastos (Table 1).

The involvement of indigenous authorities in vaccination logistics varied but remained significant. In the case of the Misak Misak and Los Pastos peoples, each *Cabildo* member was allowed to independently decide whether to get vaccinated. However, the authorities shared the information they received from health institutions such as the District Health Secretariat of Bogotá and even coordinated specific health campaigns for their communities (one day for Misak Misak and several days for Los Pastos). In contrast, Wounaan people authorities stated that each community member was free to decide for themselves; however, they also expressed distrust regarding the vaccine, thus aligning with their lower vaccination rate than other groups. Vaccination campaigns, outreach efforts, and shared information contributed to higher vaccination rates (Table 2). However, regarding vaccination information, neither individual interviews nor sharing circles mentioned target communication strategies for the indigenous population or the use of indigenous languages.

**Table 2:** Logistics, reasons, barriers, and alternatives associated with COVID-19 vaccination in Misak Misak, Wounaan, and Los Pastos peoples in Bogotá.

Category	Code	Misak Misak	Wounaan	Los Pastos
Logistics	Authorities’ attitude toward vaccination	We wanted our people [of the Misak Misak community] to decide for themselves and learn from society because it is not right to exert such a huge influence. [The people of the Misak community] could decide yes or no [to vaccination] and not to have our body directed or administered by external agents [Western healthcare system]. Instead, individuals and families [of the Misak Misak community] should become aware and conscious of the situation [about vaccination] (Misak Misak woman).	We [the authorities] explained our arguments to the community. So, what do you trust [ancestral medicine vs. vaccination]? Do you trust the [ancestral] medicine or vaccines? And if COVID arrived, there’s no problem, we would cure them again and again [with ancestral medicine] (Wounaan man).	The primary healthcare team [including ancestral health practitioners and healers] was taking care of health [before]; so, they were in charge of those topics [COVID]. They were leading along the *Cabildo* chief to motivate people to take care of themselves and to try to follow these biosafety norms (Los Pastos man).
	Vaccination days	We [the Misak Misak *Cabildo*] were called to organize a vaccination day in the Casandra neighborhood and downtown [... And also] there is a good relationship with the mayor’s office. So, they [the mayor’s office] would call us to inform us; they would say “There is a vaccination day, on this date and at this place,” and then we would inform the community in case they wanted to get vaccinated, so that they could attend the campaign (Misak Misak man).	I would often get an email saying that I had a vaccination appointment [From healthcare authorities]. One day I thought to myself: “Should I go?” and my mates told me that “we would meet at the Movistar Arena to get the [vaccine] shot.” Then, another mate told me: “I did go, but when I was about halfway in the line I got out and did not get the vaccine [...] ever.” It scared me a lot. I thought about it and said I am going to go, but at the end I have not gone [...] I am just not convinced about it (Wounaan woman).	Vaccination days were scheduled in which the entire community [Los Pastos] could participate; the call was made, and those who were going to attend were registered (Los Pastos man).
	Inter-institutional relations	I would tell them “If you have a fracture, I am not going to heal you; you have to go to the hospital. If you need surgery, I am not going to heal you with a sacred plant; there are things that can be done from here and others that can be done from there” (Misak Misak woman).	We decided [the *Cabildo*’s leaders] that the community would not get vaccinated and they, as an institution [Health authorities], respected the [Wounaan] community, because of our cosmovision and beliefs, which they [Health authorities] considered respectable (Wounaan man).	The [public] institutions also provided some support such as food packages. At the time, support was directed toward helping the most vulnerable families among the Los Pastos people, who were facing severe food insecurity (Los Pastos man).
Information	Community information	As *Cabildo* leaders, we recorded short videos in our language; that was one of the strategies we used to communicate with our people (Misak Misak woman).	We have a WhatsApp group, and we use this network; we had it before the pandemic and [In the pandemic]. We always spread the word about taking care of yourself and not going out in the street. [Those] recommendations via the WhatsApp group were an effective tool (Wounaan man).	Recordings and video clips were shared with us via WhatsApp to educate people and provide guidance on what actions to take and what to avoid (Los Pastos man).
	Infodemic	[Someone told me] you should not get vaccinated because more people are dying from the vaccine [than without it]; healthy people as well [dying]. I was also afraid because I saw on the news that those who were vaccinated the most were the ones who died the fastest (Misak Misak woman).	[The pharmaceutical company behind one of the COVID-19 vaccines] causes many [side effects] when people get vaccinated; it has even killed individuals, and many have died. I have heard that millions have died from that vaccine.	I am against vaccination because there are so many things that are said about vaccines (Los Pastos woman).
Perceptions	Vaccine side- effects	But I got the vaccine, I started to get the flu, I had a fever, every week I got sick, and I realized that it was because of the vaccine, because in our community they said the same thing; they said that they were fine before getting the vaccine, they never got sick, not even the flu, but when they got the first or second dose, it was like all diseases got stronger, even if it was the flu, it was stronger (Misak Misak woman).	We [the community] decided not to vaccinate because this medicine has [negative] effects on the human immune system, and we believe that these [negative effects] can always happen (Wounaan man).	To be honest, I had a really hard time with it [the vaccine]. The symptoms [side effects] lasted more than 21 days, more like 23 days, and after that I had a change in taste, everything tasted like onions to me; it was crazy. [It lasted] for about 4 months (Los Pastos man).
	Distrust of the vaccine development process	That is why I told them [family] not to get vaccinated [against COVID-19] because there are not enough studies, and I think that the trials they did with the vaccine should not have been done in that [short period of] time; they should have looked for other alternatives (Misak Misak man).	There was a rumor that there was something wrong with the [COVID] vaccines […] That it was going to control our minds and, well, that was one [rumor], and then the next day there was another rumor (Wounaan man).	I am a little suspicious because they did things [vaccine development] very fast; Up to that moment they kept on giving the vaccines, and people kept on dying, so I do not think it was very effective (Los Pastos man).
	Distrust of vaccine effectiveness	[...] I do not distrust the vaccine entirely. Despite the media coverage [...], I obviously thought, deep down, what will happen, what will happen to me, how will my body change after I apply a liquid [the vaccine] there? [Questions asked by the interviewee] (Misak Misak woman).	If it were to have a real effect, one runs to get vaccinated, but sometimes one hears rumors […] I think they are not doing anything (Wounaan man).	[COVID] Vaccines do not work, they really do not work. It is just a way for the government to spend [money] (Los Pastos woman).
	Cosmovision	Well, as an indigenous community [member], I told them [schoolteachers] that my daughter was not sick [...]. But they told us that she had to get vaccinated because of this and whatnot. And then the [school] principal told me that it was our decision [responsibility] if she got sick or something. But if we vaccinated her, it was okay. But then I signed [a dissent], I said “no,” I, as an indigenous community [member], know what I am doing and I am going to sign so that she does not get vaccinated (Misak Misak woman).	We can make our own decisions because we are autonomous in the way we practice [ancestral] medicine, and that is respectable and protected by law (Wounaan man).	So, I believe in cosmogony and cosmovision that big pharmaceutical houses have brought the diseases and have themselves brought the cures (Los Pastos woman).
Reasons for vaccination	Required by institutional regulations	But believe me, if I were not working, I would never have been vaccinated (Misak Misak woman).	But I also said, well, if I do not get the vaccine shot, they will not give me a job; they will take it away from me (Wounaan man).	My family [got vaccinated] because they were obligated by their jobs and had to present a vaccination card, so they got vaccinated (Los Pastos woman).
	Integration of Western (biomedical) and ancestral medicine	Well, I was just saying that I think scientific knowledge is also very important, even if it comes from outside our own [knowledge system]. They [Western and ancestral medicine] should complement each other, right? I understood it that way because you [healthcare workers] were also saving many lives over there (Misak Misak woman).	I personally would use both [Western and ancestral medicine]. I would never go against Western medicine or ancestral medicine; both should be used. I feel that ancestral medicine is useful for some things and Western medicine is useful for others (Wounaan man).	So, in our [original] territory, we combine Agüepanela [unrefined whole cane sugar juice] with lemon and ginger. However, we also take acetaminophen and combine both medicines [Western and ancestral medicine]. So, practically, what I did [Got the vaccine] is a reflection of that (Los Pastos man).
Alternatives to vaccination	Ancestral medicine	Traditional health practitioners and healers realized that there were certain plants that could repel, or could, let’s say, deal with this type of problem [COVID-19], and they made their own medicines and were already distributing them, in almost all departments where the Misak Misak community was (Misak Misak man).	It was not an obligation for us; it was not mandatory to get vaccinated. We already had our “kind of vaccine,” it was [ancestral] medicine, from medicinal plants; we did not die with that [ancestral medicine], we healed quickly, and everything was healthy almost in 15 days, after one or a few weeks everything was healthy (Wounaan man).	When you have your own [ancestral] medicine, I think you do not have to rely on other medicines. That is what your own medicine is for (Los Pastos woman).
	Western medicine (Conventional biomedicine)	[Regarding conventional medicine preventive measures against COVID-19] What we did was to guide our people on how they should behave and take care of themselves as our people, in our homes, and there were many people who managed to understand, and above all, comply with the guidance (Misak Misak woman).	It does not mean that Western medicine [Conventional biomedicine] is bad; it is not like that for us; we walk the paths of both medicines. The thing is that we know when to turn to Western medicine and when to turn to our own [ancestral] medicine (Wounaan man).	I sometimes think [in the pandemic] we were more oriented toward Western medicine [Conventional biomedicine] because it was most accessible to the community (Los Pastos man).

Among the three indigenous peoples, one of the most common opinions expressed during interviews and sharing circles was the value of ancestral medicine, which led them to prefer medicinal plants and ancestral rituals specific to their culture and territory of origin, rather than Western or conventional medicine. These methods were used to prevent and treat disease (Table 2). Ancestral rituals, medicinal plants, and other elements of ancestral medicine were considered alternatives to vaccination given their preventive and curative role (Table 2).

Furthermore, distrust in the COVID-19 vaccine was observed among the three indigenous peoples. The way in which the vaccine was developed generated a sense of distrust among respondents. In this sense, they believed that the development timeline for the vaccine was too short and that very few studies were conducted in this regard. In addition, among other rumors associated with the development of the vaccine, they believed that people were being experimented on and that the vaccines included elements that could control people (Table 2).

Negative opinions about the effectiveness of the vaccine were also expressed due to the belief that it could not prevent the disease and that those who were vaccinated could still die or were at higher risk of dying. Information about the vaccine was obtained mainly through social networks and conversations with other people. However, there was no reference by the participants to information from the health authorities that was specifically developed for indigenous people or that considered their traditions and customs. Themes associated with skepticism and distrust of the healthcare system were common, which partly contributed to the decision to not get vaccinated (Table 2).

Regarding barriers to vaccine access, participants reported no major geographical, transportation, availability, or cost constraints. The national immunization plan appeared to have functioned effectively in logistical terms. Furthermore, the Los Pastos and Misak Misak communities organized vaccination days in coordination with the District Health Secretariat. On a different note, a member of the Misak Misak community mentioned limitations in (information and communication technology) access, which affected their ability to obtain information about vaccination, as most details about vaccination days were shared via WhatsApp (Table 2). Participants who were vaccinated did not report any specific cases of discrimination related to the vaccination process. However, a few instances of discrimination within the health system were mentioned, although they were not related to COVID-19 care or vaccination.

One of the main reasons for vaccination was that educational institutions and employers had made vaccination mandatory to resume activities. The need to preserve one’s job, resume studies, or obtain access to services were the major decisive factors when considering receiving at least one dose of the vaccine, despite the participants’ opposing beliefs and desires. The factors influencing the nonacceptance of vaccination included distrust in the effectiveness of the vaccine, the use of ancestral medicine as an alternative way to cope with the disease, and the existence of rumors and contradictory information about COVID-19 vaccine. Additionally, we found that the three indigenous peoples practiced isolation measures and hand washing, regardless of their choice of using the vaccine.

## Discussion

Our study provided information on the COVID-19 vaccination experiences of the Misak Misak, Los Pastos, and Wounaan indigenous peoples living in Bogotá during the COVID-19 pandemic. Results revealed that the percentage of vaccination was heterogeneous among the three communities, and the main reason for vaccination in two communities was that it was required by work and educational institutions to resume their activities. The main reason for not being vaccinated was distrust in the vaccine. Moreover, the results showed that, in these communities, traditional medicine became an alternative treatment, which the communities perceived to be safer than the COVID-19 vaccine. Finally, infodemic was one of the main barriers against vaccination for these peoples. This study allows us to analyze the vaccination experiences of indigenous peoples living in the city and its influencing factors.

Different vaccination rates were observed among the three indigenous peoples. Although the Los Pastos and Misak Misak peoples achieved high rates of vaccination against SARS-CoV-2, 97.3% and 85.1%, respectively, the Wounaan had a coverage of 38.5%. Similar studies conducted with indigenous peoples in Brazil, Mexico, and Ecuador also reported variability in vaccination coverage across different indigenous groups or territories inhabited by them. Consistent with our study, cultural factors have been reported to influence vaccination rates [16,37,38]. Given this heterogeneity, understanding the reasons behind each *Cabildo* member’s decision to get vaccinated or not is particularly important.

In our study, the main reason for vaccination among the Misak Misak and Wounaan people was that they felt obligated to get vaccinated because of their jobs or educational institutions. This inability to decide on their own has also been described in studies conducted with other types of historically racialized minorities, particularly in the United States. Similar to the indigenous migrant population in Bogotá, Latin and African American communities fear losing their jobs or being deported for not following regulations during the pandemic [39,40]. Conversely, in the case of the Los Pastos peoples, the main reason for vaccination was to protect their health or their family or community’s health. This is consistent with findings among indigenous peoples in Canada, who chose to get vaccinated because they considered it essential to protect themselves and their communities, to resume their social activities, and to fulfill communal responsibilities [14]. Further studies are required to elucidate the factors that explain the difference between these two views in historically racialized or vulnerable groups.

The main reason for not getting vaccinated was distrust in the COVID-19 vaccine development process and fear of adverse effects. These reasons have also been identified in studies conducted with indigenous peoples from Colombia, Mexico, and Ecuador [37,41,42] as well as the majority population in Latin America and North America [40,43]. Distrust in vaccines in these communities and in general population poses major challenges to vaccination efforts, hindering acceptance of vaccines as a preventive measure against diseases. Skinner et al. conducted a study in Guatemala and described the interrelationship between myths, misinformation, and mistrust of vaccines, which provide perspectives such as corrupt intent (“vaccines are designed to kill”) or fear of what people call deadly vaccine side effects. All these factors contribute to the barrier against the vaccination process in communities [8,44].

One alternative to COVID-19 vaccination among the indigenous peoples who participated in this study was ancestral medicine. Additionally, indigenous households treated the disease by integrating their own medicine with less invasive aspects of Western medicine, such as over-the-counter medicines, isolation, use of face masks, and hand washing. This finding is consistent with two previous studies: one conducted in Putumayo with the Zio Bain indigenous people [20] and the another with the Tsáchilas indigenous group, including Otongo, Mapali, and Postea people in Ecuador and Chile [45]. In all cases, the indigenous peoples combined the use of ancestral medicinal plants, Western medicine, and isolation measures. Among the Wayuú people in Colombia, a communication guideline on Western preventive measures was also identified [46]. This evidence confirms that there is no absolute resistance to Western medicine but a complementarity that generates considerable cultural value and greater credibility among the communities.

This study assumes that, for the three indigenous communities involved, the findings were influenced by the quality and type of information received by the participants, which was marked by infodemic. This phenomenon is defined as “excessive amount of information, whether correct or incorrect, which makes it difficult for people to find reliable sources and trustworthy guidance when needed” [47]. The problem was not necessarily related to the lack of communication about COVID-19 vaccine but to the lack of effective communication among hundreds of messages, none of which was specifically directed at indigenous peoples or followed their customs and traditions. This resulted in misinformation spreading among the Misak Misak, Wounaan, and Los Pastos people, deepening their fear and mistrust regarding the COVID-19 vaccine effects, its development, and its effectiveness. This effect was identified in several studies in the majority population; specifically, two of them were conducted in Latin America and found that fake news spread orally and that ICT elicited skepticism toward the vaccine, limiting the advances of COVID-19 vaccination in these countries [16,17].

Infodemic was intensified in the case of indigenous people. A literature review found that the lack of culturally adapted information in their own language intensified the rejection of the vaccine [8]. A study identified that social networks and multimedia environments can favor vaccine acceptance only if adapted as intercultural tools [15].

Conversely, we found a difference between the three peoples who participated in this study and other indigenous peoples in terms of access to the vaccine. The Misak Misak, Wounaan, and Los Pastos peoples in Bogotá had access to vaccination campaigns and days as much as the other inhabitants of the city. Moreover, the Los Pastos people coordinated a specific vaccination day for themselves. In this case, living in the city may have favored access to the vaccine, as opposed to most indigenous people who lived in rural areas and struggled to get vaccinated against COVID-19 because of geographical restrictions [12].

Among the major limitations of this study was the limited information on vaccination in indigenous peoples living in Bogotá, making it difficult to compare the information provided by governmental entities with the information obtained in this study. Furthermore, cultural and linguistic differences between researchers and the communities may have affected the transcription process and the full understanding of the data collection methods used.

## Conclusions and recommendations

Most participants in this study were forced to get vaccinated against COVID-19 by educational, work, and/or government institutions, preventing vaccination from being autonomous, voluntary, and conscious process. Moreover, acceptance or rejection of the vaccine was strongly influenced by the infodemic, which emerged as one of the main barriers to building trust in the COVID-19 vaccine among indigenous populations, leading to doubts about its development and adverse effects.

There are many reasons why indigenous people choose to get vaccinated or not. A common and well-known barrier for those living in rural areas, where most of the world’s indigenous people reside, is limited access to health services and vaccination centers. However, the situation of the communities participating in this study did not appear to be driven by these reasons. Instead, we observed other issues noted in academic literature, such as distrust in the safety and effectiveness of the vaccine and skepticism toward health systems. This constitutes a barrier against vaccination processes in many indigenous communities [48,49]. Notably, no direct cases of discrimination were reported during the vaccination process, prompting questions about why discrimination was not observed in these communities and highlighting the need for further investigation.

Finally, our findings suggest that vaccination programs should be culturally adapted and that healthcare personnel should show the necessary cultural acumen in this regard [50,51]. Recognizing cultural identity allows the provision of quality care regardless of the context of each community. This makes it easier to address the values and beliefs of the communities, improving communication and helping deal with infodemic. Tools such as vaccination campaigns in communities’ own languages and participation of cultural intermediaries to target each intervention address infodemic and help achieve culturally safe health practices.
